# Asymmetric dynamic risk transmission between financial stress and monetary policy uncertainty: thinking in the post-covid-19 world

**DOI:** 10.1007/s11156-023-01140-9

**Published:** 2023-03-11

**Authors:** Chao Liang, Yanran Hong, Luu Duc Toan Huynh, Feng Ma

**Affiliations:** 1 School of Economics and Management, Southwest Jiaotong University, Chengdu, 610031 China; 2 School of Mathematics, Southwest Jiaotong University, Chengdu, 610031 China; 3 School of Business and Management, Queen Mary University of London, London, United Kingdom

**Keywords:** Financial stress, Monetary policy uncertainty, Granger-causality test, Asymmetric, Time-varying, C32, F39, G11

## Abstract

Considering the dramatically increasing impact of the COVID-19 outbreak on monetary policy and the uncertainty in the financial system, we aim to examine the dynamic asymmetric risk transmission between financial stress and monetary policy uncertainty. Our sample covers 30 years of data. We first employ the conventional Granger causality test to examine the average relationship between financial stress and monetary policy uncertainty, and the results cannot provide evidence of causality between them. However, from an asymmetric perspective, we further detect the strongly apparent existence of the asymmetric structure of causality between them. Finally, we conduct further research on the asymmetric impacts from a time-varying perspective. The time-varying test finds that this relationship can be influenced by major events, especially the dot-com bubble, the 2009 financial crisis, and the current COVID-19 pandemic. Thus, one can learn more information about the influencing mechanism between financial stress and monetary policy with our work, which may be beneficial for making better decisions in the future.

## Introduction

Undeniably, maintaining financial stability has been regarded as the most vital duty of policy-makers for a long time. Therefore, during periods of high financial instability,^1^ policy-makers implement corresponding economic policies, especially monetary policy, to cope with risks originating from financial markets (Rigobon & Sack 2003; Stein 2012; Liang et al. 2021). The central bank plays a key role in maintaining this stability by mainly smoothing changes in the exchange rate (Schinasi 2003). However, this behaviour has been verified to bring some basic risks for financial markets (Goodfriend 1987; Phan et al. 2021). Meanwhile, most financial banks may widely conduct hedging due to their vulnerability to exchange rate changes (Choi et al. 1992). Financial institutions are also likely to choose various tools to hedge these risks. These hedges can lead to residual effects on the financial system. This kind of effect, combined with the above-mentioned risks originating from the central bank and other underlying risks, will in turn constrain the implementation of monetary policy (Driffill et al. 2006). On the other hand, Guo et al. (2022) pointed out that policy changes to tackle a crisis in the short term will mean increased policy uncertainty, which may lead to more negative and bad results. In the last 30 years, multifarious extreme and sudden episodes have strongly hit the stability of the financial system. In such a case, banks, institutions, and individuals can take steps to minimize the losses stemming from financial risks if we can detect whether and how financial instability and monetary policy uncertainty affect each other.

Recently, various risks have become intertwined in this post-COVID-19 period. Many scholars have investigated the relationship between financial fundamentals in the COVID-19 period (Goodell 2020; Szczygielski et al. 2022; Yarovaya et al. 2022). In particular, Wei and Han (2021) studied the effect of the COVID-19 outbreak on risk transmission from monetary policy to financial markets and found that this crisis has weakened monetary policy transmission. They argued that stronger unconventional monetary policies are more effective in the post-COVID-19 period. Padhan and Prabheesh (2021) revealed the adverse effects of COVID-19 on the economy by considering monetary policy, macroprudential policy, and fiscal policy. Their work suggested that more policy coordination at the national and international levels is needed to address this negative impact. These studies offer clear implications for policy-makers to adjust monetary policies in the post-COVID-19 period. However, they have ignored the impact of other major events on the relationship and have not explored whether there is a two-way effect between them. Which is likely to be the main driver? As mentioned above, short-run and shifting changes in monetary policy could lead to more uncertainty. Much attention has been paid to this uncertainty for a long time (Stulz 1986; Bekaert et al. 2013; Husted et al. 2020). With increasingly complex and fickle financial conditions, the linkage (risk transmission) between financial instability and monetary policy uncertainty seems to be overlooked. Therefore, in the context of COVID-19, this paper further examines the bidirectional relationship between financial stress and monetary policy uncertainty from an asymmetric and dynamic perspective. In this paper, we conduct a survey by employing and extending the Granger causality framework. In summary, our contributions are as follows.

Different from existing works, we mainly apply a news-based monetary policy uncertainty index to capture risk from monetary policy changes. We focus on the US financial market. Thus, we consider the US monetary policy uncertainty index (MPU) to capture changes in the implementation of monetary policy, and we adopt the Kansas City Financial Stress Index (KCFSI) to represent the dynamics of financial uncertainty in the US financial market.^2^ Although many studies have long explored the linkage between them through different economic and statistical tools (Herrero & Del Rio 2004; Stein 2012; Adrian & Liang 2016; Bratis et al. 2021; Hu et al. 2022), there are some research gaps. A large branch of the literature mainly discusses the influencing channel between financial conditions or monetary policy and various financial assets (Sims & Zha 2006; Kang & Ratti 2013; Liang et al. 2020; Hong et al. 2022a). On the other hand, limited attention has been paid to the direct impact of monetary policy on financial stress and the response of monetary policy to financial stress. By directly observing the spike in historical fluctuations, Cardarelli et al. (2011) found that financial stress seems to be closely linked to monetary policy. Baxa et al. (2013) examined the dynamic correlation coefficient to briefly detect whether and how financial stress affects US monetary policy. Therefore, this paper contributes to research on financial stress and monetary policy by applying the FSI and MPU.

According to our investigation, this paper is the first to explore the causal relationship between the FSI and MPU. Specifically, we not only examine their average causality but also detect the asymmetric characteristic of this relationship with the asymmetric Granger causality test of Hatemi-j (2012). The Granger causality test (Granger 1969), which is a flexible, effective, and powerful method for investigating the potential connections between time series, mainly identifies causality by assuming that the underlying variables behave as average causality does. In addition, the test is widely used in econometric research because it detects a directional relationship between series, known as the transmission of risk (Freeman 1983; Ding et al. 2006; Lopez & Weber 2017). Hatemi-j (2012) extended this conventional method, and the extension can investigate asymmetric effects among sequences and clarify the influencing mechanism between financial fundamentals in more detail. This method first divides financial fundamentals into two components, namely, negative and positive shocks, and it further proceeds to test the causality between pairwise shocks. More importantly, it has received much attention and has been applied in many research fields, such as medical treatment, tourism, and energy (Sebri & Ben-Salha 2014; Aslan 2016; Wen et al. 2022). Thus, based on Hatemi-j (2012), we divide both the FSI and MPU into different shocks (each positive and negative) and examine the causality between each FSI and MPU shock.

Moreover, to observe the dynamic changes in their causality, we employ the asymmetric causality test extended by Hong et al. (2022b). A great number of studies emphasize the importance of the time-varying characteristic (Song et al. 2011; Ajmi et al. 2015; Ushio et al. 2018). Hong et al. (2022b) combines the asymmetric causality test with the three procedures of Shi et al. (2020) to develop a novel time-varying asymmetric Granger causality test that enables us to detect changes in the asymmetric relationship between the FSI and MPU. With the rapidly rising integration and globalization among financial markets, systemic financial risk has also been increasing at a staggering speed (Silva et al. 2017). During periods of crisis and turbulence, such as the Asian financial crisis, the dot-com bubble, the 2008 financial crisis, the Russia-Ukraine conflict, and the persistent COVID-19 pandemic, financial stress appears to be higher than normal, posing a challenge to market participants (Bai et al. 2021). Clearly, if we focus only on static relationships and information, the impact originating from certain extreme events may be underestimated, which may further lead to misjudgements about current market conditions. Thus, to accurately track market fluctuations, we should pay close attention to the time-varying characteristic of the asymmetric causality between the FSI and MPU. In particular, we can also conduct the asymmetric time-varying causality test for these pairwise shocks with the forward recursive, rolling, and recursive evolving procedures.

Hence, we propose the following questions and aim to answer them. First, is there a significant causal relationship running from the FSI to MPU? Can negative and positive FSI shocks affect MPU shocks in the same way? Conversely, is there a significant causal relationship running from MPU to the FSI? Can negative and positive MPU shocks affect FSI shocks in the same way? Furthermore, how do these asymmetric impacts of FSI shocks on MPU shocks and responses of FSI shocks to MPU shocks change over time?

The rest of the paper is structured as follows. Section 2 introduces the method that we applied and extended. Section 3 explains the data selection and presents the descriptive statistics. We display the empirical results in Sect. 4. In Sect. 5, we discuss our results, and in Sect. 6, we conduct a supplementary analysis. Finally, Sect. 7 concludes.

## Methodology

### Asymmetric causality test

The conventional causality test can provide one-shot and static results for the causality between the FSI and MPU, but it overlooks the asymmetric characteristic of financial shocks (Granger 1969, 1980). To test the asymmetric effect, that is, whether different causalities may exist in positive and negative shocks, Hatemi-j (2012), motivated by the work of Granger and Yoon (2002), extended the conventional Granger causality test and thus proposed the asymmetric Granger causality test. In this paper, we focus on the causality between the FSI and MPU; thus, we assume that the FSI is represented by ${F}_{t}$ and that MPU is represented by ${M}_{t}$. Based on Granger and Yoon (2002), ${F}_{t}$ and ${M}_{t}$ can be expressed as follows:

1 $${F}_{t}={F}_{t-1}+{\varepsilon }_{1t}={F}_{0}+{\sum }_{i=1}^{t}{\varepsilon }_{1i}$$

2 $${M}_{t}={M}_{t-1}+{\varepsilon }_{2t}={M}_{0}+{\sum }_{i=1}^{t}{\varepsilon }_{2i}$$

where $t=1, 2,\dots ,T$. ${F}_{0}$ and ${M}_{0}$ are the initial values of ${F}_{t}$ and ${M}_{t}$, respectively. ${\varepsilon }_{1i} \sim NID\left(\mathrm{0,1}\right)$ and ${\varepsilon }_{2i} \sim NID\left(\mathrm{0,1}\right)$. Inspired by Hatemi-j (2012), positive and negative shocks can be defined as follows:

3 $$F:\varepsilon_{1i}^{ + } = {\text{max}}\left( {\varepsilon_{1i} ,0} \right),\varepsilon_{1i}^{ - } = {\text{min}}\left( {\varepsilon_{1i} ,0} \right)$$

4 $$M:\varepsilon_{2i}^{ + } = {\text{max}}\left( {\varepsilon_{2i} ,0} \right),\varepsilon_{2i}^{ - } = {\text{min}}\left( {\varepsilon_{2i} ,0} \right)$$

Thus, ${\varepsilon }_{1i}={{\varepsilon }_{1i}}^{+}+{{\varepsilon }_{1i}}^{-}$ and ${\varepsilon }_{2i}={{\varepsilon }_{2i}}^{+}+{{\varepsilon }_{2i}}^{-}$, implying that

5 $${F}_{t}={F}_{0}+{\sum }_{i=1}^{t}{\varepsilon }_{1i}={F}_{0}+{\sum }_{i=1}^{t}{\varepsilon }_{1i}^{+}+{\sum }_{i=1}^{t}{\varepsilon }_{1i}^{-}$$

6 $${M}_{t}={M}_{0}+{\sum }_{i=1}^{t}{\varepsilon }_{2i}={M}_{0}+{\sum }_{i=1}^{t}{\varepsilon }_{2i}^{+}+{\sum }_{i=1}^{t}{\varepsilon }_{2i}^{-}$$

Based on the above-mentioned definition, positive and negative shocks of each variable^3^ can be simply introduced as ${F}^{+}={\sum }_{i=1}^{t}{\varepsilon }_{1i}^{+}$, ${F}^{-}={\sum }_{i=1}^{t}{\varepsilon }_{1i}^{-}$, ${M}^{+}={\sum }_{i=1}^{t}{\varepsilon }_{2i}^{+}$ and ${M}^{+}={\sum }_{i=1}^{t}{\varepsilon }_{2i}^{+}$. Then, causality between pairwise shocks can be detected. For instance, we begin our research by testing the null hypothesis of noncausality from ${M}_{t}^{+}$ to ${F}_{t}^{+}$. Based on Kilian and Park (2009), the null hypothesis can be examined by the *p*-order VAR model:

7 $$\left(\begin{array}{c}{F}_{t}^{+}\\ {M}_{t}^{+}\end{array}\right)=\left(\begin{array}{c}{\alpha }_{10}\\ {\alpha }_{20}\end{array}\right)+\left(\begin{array}{cc}{\alpha }_{11}& {\gamma }_{11}\\ {\alpha }_{21}& {\gamma }_{21}\end{array}\right)\left(\begin{array}{c}{F}_{t-1}^{+}\\ {M}_{t-1}^{+}\end{array}\right)+\dots +\left(\begin{array}{cc}{\alpha }_{1p}& {\gamma }_{1p}\\ {\alpha }_{2p}& {\gamma }_{2p}\end{array}\right)\left(\begin{array}{c}{F}_{t-p}^{+}\\ {M}_{t-p}^{+}\end{array}\right)+\left(\begin{array}{c}{\varepsilon }_{1t}\\ {\varepsilon }_{2t}\end{array}\right)$$

If ${M}_{t}^{+}$ does not Granger cause ${F}_{t}^{+}$, then ${\gamma }_{ki}=0 (k=1, 2, i=1, \dots , p)$. In this case, one can select the lag order *p* by the HJC information criterion and further test the null hypothesis by the Wald test.^4^ Moreover, considering the potential non-asymptotic property of the small-sample distribution in the Wald test and the multivariate normality and ARCH effect of financial fundamentals, a bootstrap simulation technique should be introduced. For more details about this method, refer to Hatemi-j (2012).

### Asymmetric time-varying causality test

To observe the dynamic changes in a causal relationship, Shi et al. (2020)_ENREF_11 proposed a time-varying causality test based on Shi et al. (2018). This method includes three bootstrap procedures, including the forward recursive, rolling and recursive evolving algorithms. Based on Shi et al. (2020), this time-varying causality test is constructed by the three algorithms to calculate the supremum of the Wald statistical sequence. Specifically, the forward recursive algorithm is based on Thoma (1994). The rolling procedure originates from Swanson (1998), and the recursive evolving algorithm is from Phillips et al. (2015a, b). Notably, subsamples of the original data are used in these three algorithms to calculate Wald statistics. In addition, since these three algorithms obtain statistical values through multiple testing, Phillips et al. (2015a) suggested that the bootstrap method should be applied to control the experience scale in the implementation process.

Motivated by the works above, Hong et al. (2022b) extend the asymmetric causality test by combining these three algorithms. Specifically, we can first define both positive and negative FSI and MPU shocks based on Eqs. (1–6). Then, based on Toda and Yamamoto (1995) and Dolado and Lütkepohl (1996), we assume a bivariate LA-VAR model. For instance, we focus on the time-varying causality between positive shocks ${(F}_{t}^{+}, {M}_{t}^{+})$:

8 $${F}_{t}^{+}={\alpha }_{10}+{\alpha }_{11}t+\sum_{i=1}^{k+d}{\beta }_{1i}{F}_{ t-1}^{+}+\sum_{i=1}^{k+d}{\gamma }_{1i}{M}_{ t-1}^{+}+{\varepsilon }_{1t}$$

9 $${M}_{t}^{+}={\alpha }_{20}+{\alpha }_{21}t+\sum_{i=1}^{k+d}{\beta }_{2i}{F}_{t-1}^{+}+\sum_{i=1}^{k+d}{\gamma }_{2i}{M}_{t-1}^{+}+{\varepsilon }_{2t}$$

where *t* denotes the time trend, *k* represents the lag order in the VAR model, and ${\varepsilon }_{1t}$ and ${\varepsilon }_{2t}$ are the error terms. *d* is the augmentation of the possible largest order of variable integration in the VAR model. If ${F}_{t}^{+}$ cannot Granger cause ${M}_{t}^{+}$, then the null hypothesis ${H}_{0}: {\gamma }_{1i}=\dots ={\gamma }_{1K}=0$ cannot be rejected. Furthermore, Eqs. (8–9) can be simply expressed as follows:

10 $${Y}_{t}^{+}={A}_{0}+{A}_{1}t+\sum_{i=1}^{k}{J}_{i}{Y}_{t-i}^{+}+\sum_{j=k+1}^{k+d}{J}_{j}{Y}_{t-j}^{+}+{e}_{t}$$

This regression equation can be rewritten as follows:

11 $${Y}_{t}^{+}={\Gamma }_{1}{\tau }_{t}+{\Phi }_{1}{X}_{t}^{+}+{\Psi }_{1}{Z}_{t}^{+}+{e}_{t}$$

where ${\Gamma }_{1}=({A}_{0}{,A}_{1})$, ${\tau }_{t}={(1,t)}^{\mathrm{^{\prime}}}$,${\Phi }_{1}=({J}_{1},\dots ,{J}_{k})$, ${X}_{t}^{+}={({Y}_{t-1}^{+},\dots ,{Y}_{t-K}^{+})}^{\mathrm{^{\prime}}}$, ${\Psi }_{1}=\left({J}_{k+1},\dots ,{J}_{k+d}\right)=0$, and ${Z}_{t}^{+}={({Y}_{t-k-1}^{+},\dots ,{Y}_{t-k-d}^{+})}^{\mathrm{^{\prime}}}$. Shi et al. (2018) suggested that the null hypothesis of noncausality is given as follows:

12 $${H}_{0}: {R}_{m\times {n}^{2}k}{\phi }_{{n}^{2}k\times 1}.=0$$

where $\phi =vec( \Phi )$ using row vectorization. To conduct the estimation more conveniently, Eq. (11) can be replaced with the following:

13 $$Y=\tau {\Gamma }^{^{\prime}}+X{\Phi }^{^{\prime}}+Z{\Psi }^{^{\prime}}+E$$

where $Y={({Y}_{1}^{+},\dots ,{Y}_{T}^{+})}^{\mathrm{^{\prime}}}$, $\tau =({\tau }_{1},\dots ,{\tau }_{T})$, $X={({X}_{1}^{+},\dots ,{X}_{T}^{+})}^{\mathrm{^{\prime}}}$, $\Phi =({\Phi }_{1},\dots ,{\Phi }_{T})$, $Z=({Z}_{1}^{+},\dots ,{Z}_{T}^{+})$, $\Psi ={\Psi }_{1},\dots , {\Psi }_{T}$, and $E=({e}_{1},\dots ,{e}_{T})$. Assume that ${Q}_{\tau }={I}_{T}-\tau {({\tau }^{\mathrm{^{\prime}}}\tau )}^{-1}{\tau }^{\mathrm{^{\prime}}}$ and $Q={Q}_{\tau }-{Q}_{\tau }Z{({Z}^{\mathrm{^{\prime}}}{Q}_{\tau }Z)}^{-1}{Z}^{\mathrm{^{\prime}}}{Q}_{\tau }$. The OLS estimator is $\widehat{\Phi }={Y}^{\mathrm{^{\prime}}}QX{({X}^{\mathrm{^{\prime}}}QX)}^{-1}$. The standard Wald statistic is as follows:

14 $$\mathcal{W}={(R\widehat{\phi })}^{^{\prime}}{[R\left\{{\widehat{\Sigma }}_{E}\otimes {({X}^{^{\prime}}QX)}^{-1}\right\}{R}^{^{\prime}}]}^{-1}R\widehat{\phi }$$

where $\widehat{\phi }=vec(\widehat{\Phi })$, ${\widehat{\Sigma }}_{E}=\frac{1}{T}{\widehat{E}}^{^{\prime}}\widehat{E}$, and $\otimes$ is the Kronecker product. The Wald statistic satisfies the asymptotic null distribution ${\upchi }_{m}^{2}$, where *m* is the number of constraints.

In line with Shi et al. (2020) and Hong et al. (2022b), we first assume the starting points and ending points of the (sub)sample in the regression estimation, namely, ${\tau }_{0}$ and ${\tau }_{b}$, respectively. Thus, the sample size of the bootstrap data is ${T}_{b}={\tau }_{0}+{\tau }_{b}-1$. Generate a bootstrapping sample in light of the above-mentioned VAR model. Then, compute the test statistics for the forward, rolling, and recursive evolving procedures.

Forward: $${M}_{1,t}^{b}={max}_{t\in [{\tau }_{0}, {\tau }_{0}+{\tau }_{b}-1]}({W}_{1,t}^{b})$$

15 $${\text{Rolling}}:M_{{t - \tau_{0} + 1,t}}^{b} = max_{{t \in \left[ {\tau_{0} ,{ }\tau_{0} + \tau_{b} - 1} \right]}} \left( {M_{{t - \tau_{0} + 1,t}}^{b} } \right)$$

Recursive: $${SM}_{t}^{b}\left({\tau }_{0}\right)={max}_{t\in [{\tau }_{0}, {\tau }_{0}+{\tau }_{b}-1]}({SM}_{t}^{b}\left({\tau }_{0}\right))$$

Next, the steps above are repeated 499 times. Finally, the critical values of these three rolling window approaches are chosen as the 95th percentiles of ${\left\{{M}_{1,t}^{b}\right\}}_{b=1}^{B}$, ${\left\{{M}_{t-{\tau }_{0}+1,t}^{b}\right\}}_{b=1}^{B}$ and ${\left\{{SM}_{t}^{b}\left({\tau }_{0}\right)\right\}}_{b=1}^{B}$. In particular, the recursive evolving window procedure is demonstrated to have the best performance. Thus, we mainly analyse the empirical results based on it. See Shi et al. (2020) for more details about this procedure.

## Data

### Data

This paper uses the Baker-Bloom-Davis (BBD) MPU index based on Access World News. The reasons for choosing the BBD MPU index are as follows. First, the indicator is constructed based on news information, which can identify the economic impact caused by changes in monetary policy and capture a broader concept than monetary policy. A large amount of literature confirms the excellent ability of the index in regard to these two aspects (Baker et al. 2016; De Pooter et al. 2021; Hu et al. 2022). Second, MPU and MPU-10 are built to the same standard. MPU-10 is mainly built based on 10 major newspapers, while the BBD MPU index is based on hundreds of newspapers and contains more information on monetary policy changes than the former. Finally, it is worth noting that there is a large difference between the Husted-Rogers-Sun (HRS) MPU index and the BBD MPU index, but the BBD MPU is more reflective of public concerns (Husted et al. 2020). Furthermore, considering data availability, we do not consider the HRS MPU index to include more recent information for analysis.^5^ Information about the data is presented in Table 1.

**Table 1:** Summary of MPU and FSIs

Full name	Abbreviation	Frequency	Size	Access
*Panel A: MPU indices*				
Baker-Bloom-Davis MPU index based on Access World News	MPU	Monthly	1985-present	http://www.policyuncertainty.com/bbd_monetary.html
Baker-Bloom-Davis MPU index based on 10 major papers	MPU-10	Monthly	1985-present	http://www.policyuncertainty.com/bbd_monetary.html
Husted-Rogers-Sun MPU index	HRS MPU	Monthly	1985–2021	https://sites.google.com/site/lucasfhusted/data
*Panel B: FSIs*				
Kansas City Financial Stress Index	KCFSI	Monthly	1990-present	https://fred.stlouisfed.org/series/KCFSI
Newspaper-based Financial Stress Indicator	FSI	Monthly	1889–2016	http://www.policyuncertainty.com/financial_stress.html
(Office of Financial Research) Financial Stress Index	OFR FSI	Daily	2000-present	https://www.financialresearch.gov/financial-stress-index/
Cleveland Financial Stress Index	CFSI	Daily	1991–2016	https://fred.stlouisfed.org/series/CFSI
St. Louis Fed Financial Stress Index	STLFSI	Weekly	1993-present	https://fred.stlouisfed.org/series/STLFSI3
Chicago Fed National Financial Conditions Index	NFCI	Weekly	1971-present	https://fred.stlouisfed.org/series/NFCI

This table summarizes the MPU indices and FSIs

On the other hand, for the purpose of capturing the uncertainty and instability of the financial system, we chose the Kansas City Financial Stress Index (KCFSI) proposed by Hakkio and Keeton (2009) to represent financial stress in US financial markets. To more conveniently explain why we chose this index, we summarize some popular US financial stress indices in Table 1. First, we focus on the sampling frequency of the data. The models constructed in this paper require the same sampling frequency; thus, the FSI that we choose can only be in monthly units due to the monthly nature of MPU. Second, we look at the length of the data. To conduct research based on more historical information, especially recent information, we choose a data sample that is as long as possible. More importantly, from the perspective of data construction, the KCFSI attempts to identify key phenomena of all possible financial crises and aims to highlight five phenomena that are closely related to changes in financial markets (Manamperi 2015). In particular, the index focuses on the increased asymmetry of information (Hakkio & Keeton 2009), which may be more conducive to our analysis of the potential asymmetric relationship between MPU and the FSI. Therefore, our work is based on the KCFSI.

**Fig. 1:**
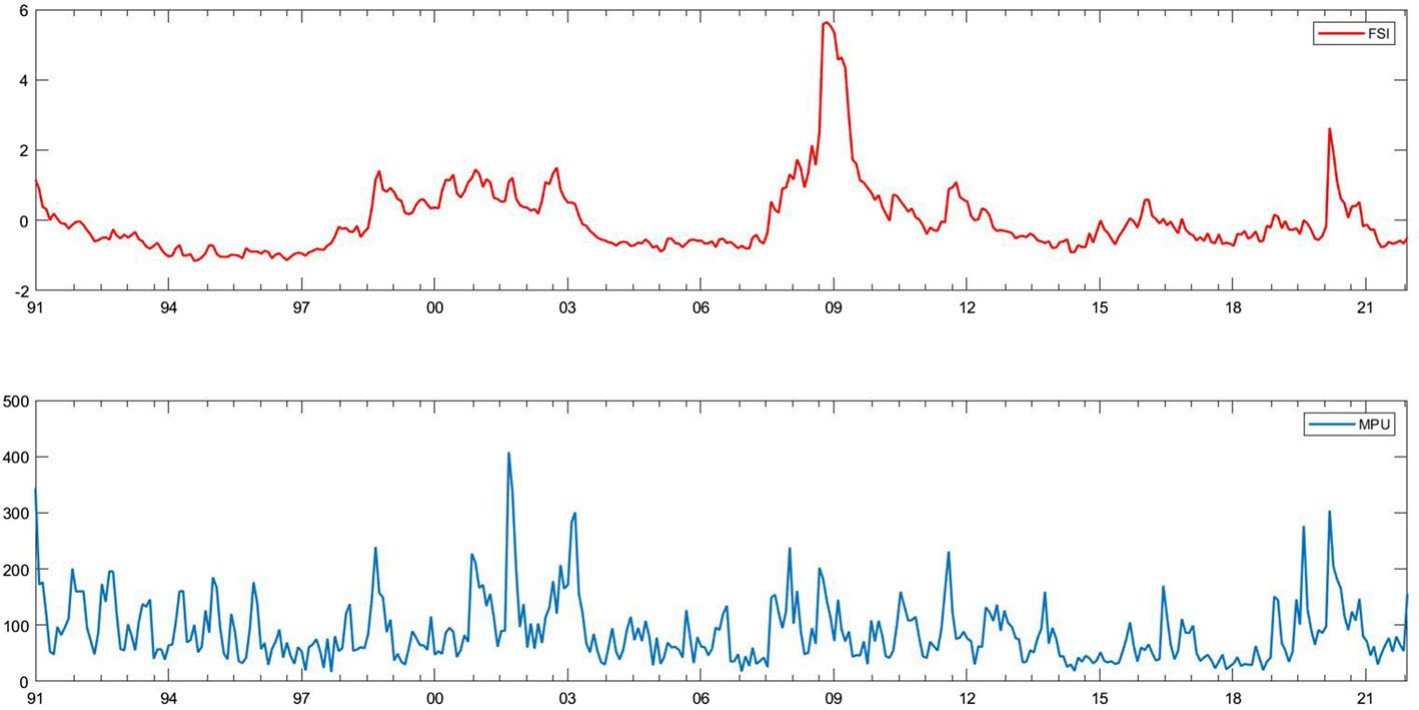
Historical fluctuations in the FSI and MPU. *Notes*: Sample period: from January 1991 to December 2021

For the reasons above, we obtain monthly sample data from January 1991 to December 2021. Table 2 displays the descriptive statistics. We calculate the maximum (Max), minimum (Min), mean, standard deviation (S.D.), skewness (Skew), and kurtosis (Kurt). The maximum of the FSI is positive, but its minimum is negative. The mean of the FSI is negative, implying that financial stress is not tightened on average. For MPU, the maximum, minimum and mean are positive. The dispersion degree of the FSI is larger than that of MPU. The FSI is right-skewed, and its kurtosis is higher than the normal distribution. Thus, it is with MPU as well. The ADF and KPSS tests show inconsistent results, suggesting that they are non-stationary. Figure 1 plots the historical fluctuations in the FSI and MPU. It can be seen that throughout the sample period, the FSI is highest during the 2008 financial crisis. However, the dramatic volatility of MPU can be seen from 2000 to 2003. In response to the bursting of the dot-com bubble, the Federal Reserve lowered the federal funds rate 13 consecutive times from January 2001 to June 2003, resulting in the real estate bubble in the United States.

**Table 2:** Descriptive statistics

	Max	Min	Mean	S.D	Skew	Kurt	JB	ADF	KPSS
FSI	5.6392	− 1.1559	− 0.0072	1.0135	2.7310	13.8153	2275.5000***	− 3.3166^***^	1.4938^***^
MPU	407.9409	16.5745	88.3520	56.7943	1.8357	8.0171	599.0888***	− 5.2103^***^	0.2220^***^

This table shows the descriptive statistics of the FSI and MPU

***denotes statistical significance at the 1% level

### Preliminary results

Before investigating the causality between the FSI and MPU, it is important to note that the Granger causality test is based on the stationary series (Granger 1969). In addition, although Hatemi-j (2012) points out that the asymmetric causality test does not require stationary series, the time-varying causality test of Shi et al. (2020) suggests that the LA-VAR model should contain more information about the maximum possible order of integration. Hence, we need to prefilter the time series by taking differences.

In particular, motivated by Wang et al. (2021), we also examine the existence of multivariate normality and ARCH effects. Table 3 shows both diagnostic test results for the VAR model. For raw data and each pairwise shock, the null hypothesis of multivariate normality is strongly rejected at the significance level of 1%. In addition, in all cases, the null hypothesis of non-multivariate ARCH can be significantly rejected. Hence, as mentioned in Hatemi-j (2012) and Wang et al. (2020b), it is crucial to adopt the bootstrapping method for further analysis.

**Table 3:** Test for multivariate normality and the ARCH effect

	M–N	M-ARCH
$(F,M)$	< 0.0001	< 0.0001
$({F}^{-},{M}^{-})$	< 0.0001	< 0.0001
$({F}^{+},{M}^{-})$	< 0.0001	< 0.0001
$({F}^{-},{M}^{+})$	< 0.0001	< 0.0001
$({F}^{+},{M}^{+})$	< 0.0001	< 0.0001

M–N and M-ARCH represent the tests for multivariate normality (Doornik & Hansen 2008) and ARCH (Scott Hacker & Hatemi-J 2005), respectively

## Main empirical results

### Asymmetric static causality

The conventional Granger causality test can provide symmetric and static results. In line with Wang et al. (2020b) and Wang et al. (2021), the bootstrapping method can be applied. We focus on critical values at the significance level of 5%. If the Wald test statistic value with the raw data is higher than that with the bootstrap data, the null hypothesis of non-Granger causality running from the FSI (MPU) to MPU (the FSI) can be rejected. Table 4 displays both symmetric and asymmetric causalities between the FSI and MPU.

**Table 4:** Conventional and asymmetric causality between the FSI and MPU

	Wald	1%	5%	10%
*Panel A: FSI ↛ MPU*				
$F\nrightarrow M$	0.8255	6.4418	4.0074	2.6080
${F}^{-}\nrightarrow {M}^{-}$	0.1536	12.3564	6.3587	4.4775
${F}^{+}\nrightarrow {M}^{-}$	33.0385***	13.0346	6.6944	4.6560
${F}^{-}\nrightarrow {M}^{+}$	2.5516	12.8069	8.3350	6.3185
${F}^{+}\nrightarrow {M}^{+}$	0.6262	12.7939	6.9505	4.9191
*Panel B: MPU ↛ FSI*				
$M\nrightarrow F$	1.8190	6.6510	3.8654	2.5663
${M}^{-}\nrightarrow {F}^{-}$	5.4175*	14.8445	7.0736	4.6722
${M}^{+}\nrightarrow {F}^{-}$	36.8991***	15.5650	8.7965	6.6566
${M}^{-}\nrightarrow {F}^{+}$	2.3080	10.8945	6.1677	4.4991
${M}^{+}\nrightarrow {F}^{+}$	0.6762	13.2088	6.5476	4.5452

Null hypotheses that are significantly rejected at the 1% and 5% significance level are shown in bold

*F* stands for the FSI, and *M* is MPU. + and - are positive and negative shocks, respectively

*, **, and *** denote rejection of the null hypothesis at the 10%, 5%, and 1% significance levels, respectively

**Q1:** Is there a significant causal relationship running from the FSI to MPU? Can negative and positive FSI shocks affect MPU shocks in the same way?

Panel A of Table 4 shows that the null hypothesis of noncausality from the FSI to MPU cannot be rejected, indicating that financial stress cannot affect uncertainty about monetary policy on average. This finding seems to be reasonable since the conventional test can provide only a one-shot result, which may not represent a true impact of the FSI on MPU. The uncertainty about and the instability of financial markets are proven to have a significant impact on monetary policy, especially in the highest spike of financial stress (Cardarelli et al. 2011). Hence, we further investigate whether an asymmetric structure exists in this impact. We find that positive FSI shocks can Granger cause negative MPU shocks at the 1% significance level, indicating that higher financial stress may lead to lower uncertainty about monetary policy. Undoubtedly, policy-makers closely track financial market movements, especially unusual movements. Hence, policy-makers will formulate various policies to stabilize the financial system when financial stress fluctuates dramatically. In this case, more attention may be paid to monetary policy, which could be less uncertain.

**Q2**: Is there a significant causal relationship running from MPU to the FSI? Can negative and positive MPU shocks affect FSI shocks in the same way?

From Panel B in Table 4, we first see that MPU does not have an impact on the FSI on average. However, the hypothesis of noncausality running from negative MPU shocks to negative FSI shocks is rejected at the significance level of 10%. Interestingly, negative FSI shocks can also be affected by positive MPU shocks at the 1% significance level. The financial system is easily influenced by stock markets, which are driven by future returns. Returns are closely connected to inflation and the output gap (Castelnuovo & Nistico 2010). Therefore, monetary policy needs to be applied to tackle these challenges. Through these efforts, financial stress may become lower.

### Asymmetric time-varying causality

**Fig. 2:**
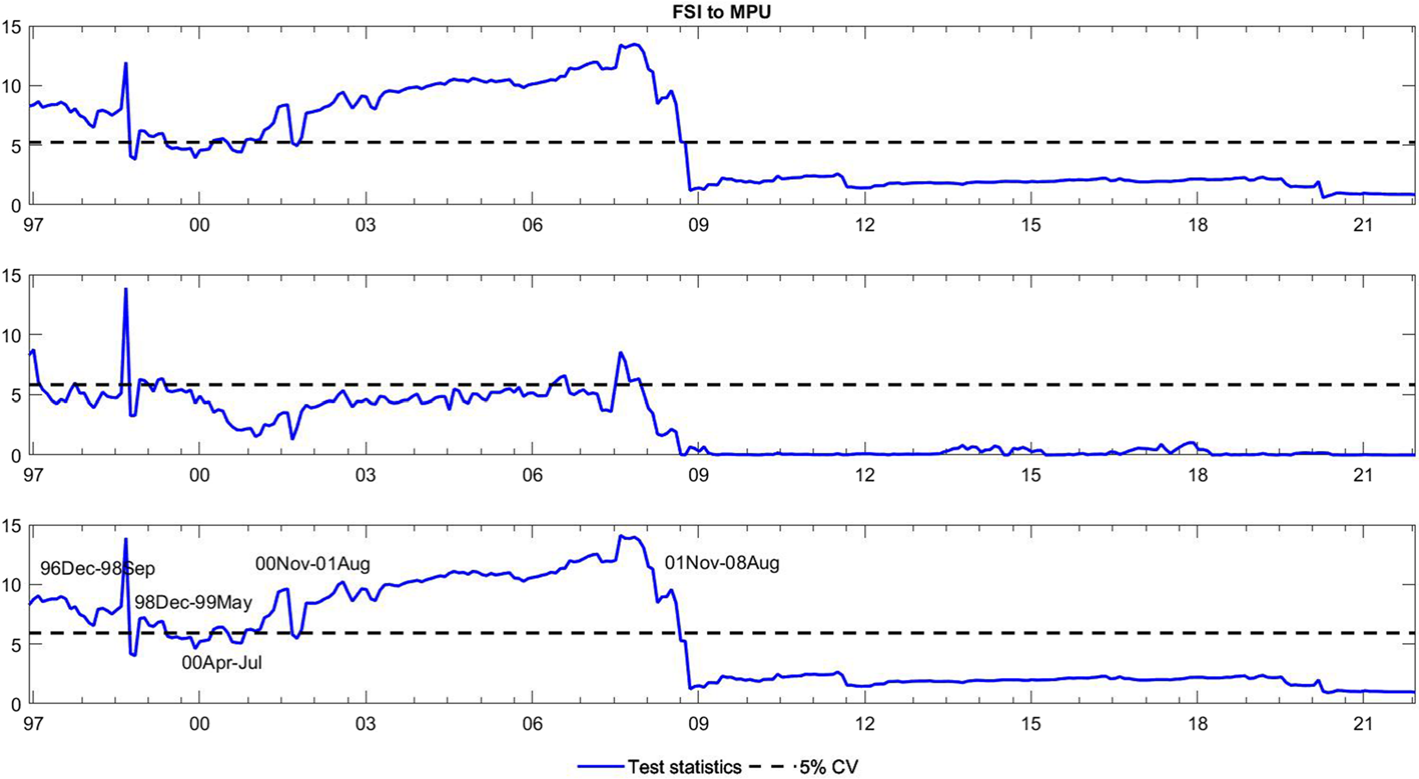
Dynamic causality running from the FSI to MPU. *Notes*: This figure displays the dynamic changes in average causality running from the FSI to MPU. If the blue line is higher than the black line, the FSI can Granger cause MPU (Color figure online)

**Fig. 3:**
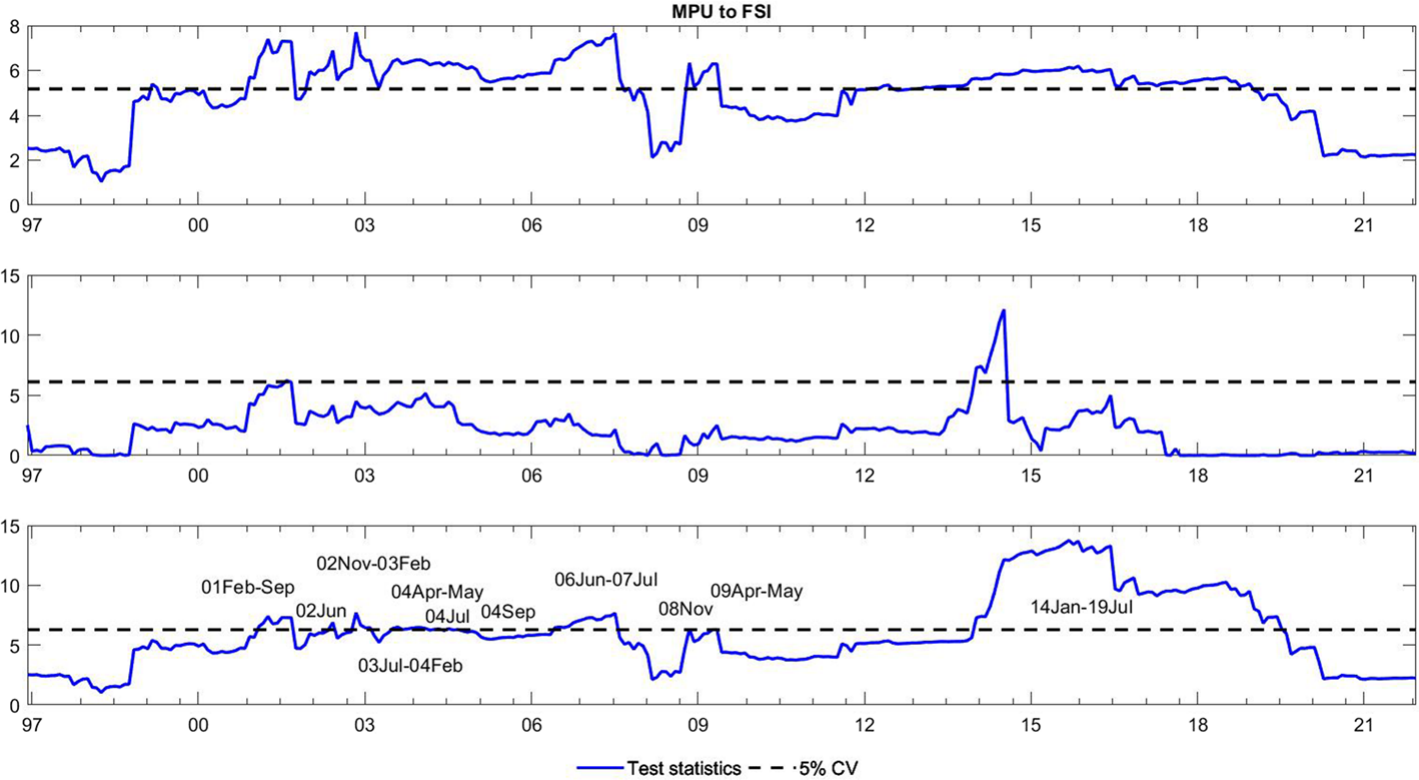
Dynamic causality running from MPU to the FSI. *Notes*: This figure displays the dynamic changes in average causality running from MPU to the FSI. If the blue line is higher than the black line, MPU can Granger cause the FSI (Color figure online)

**Fig. 4:**
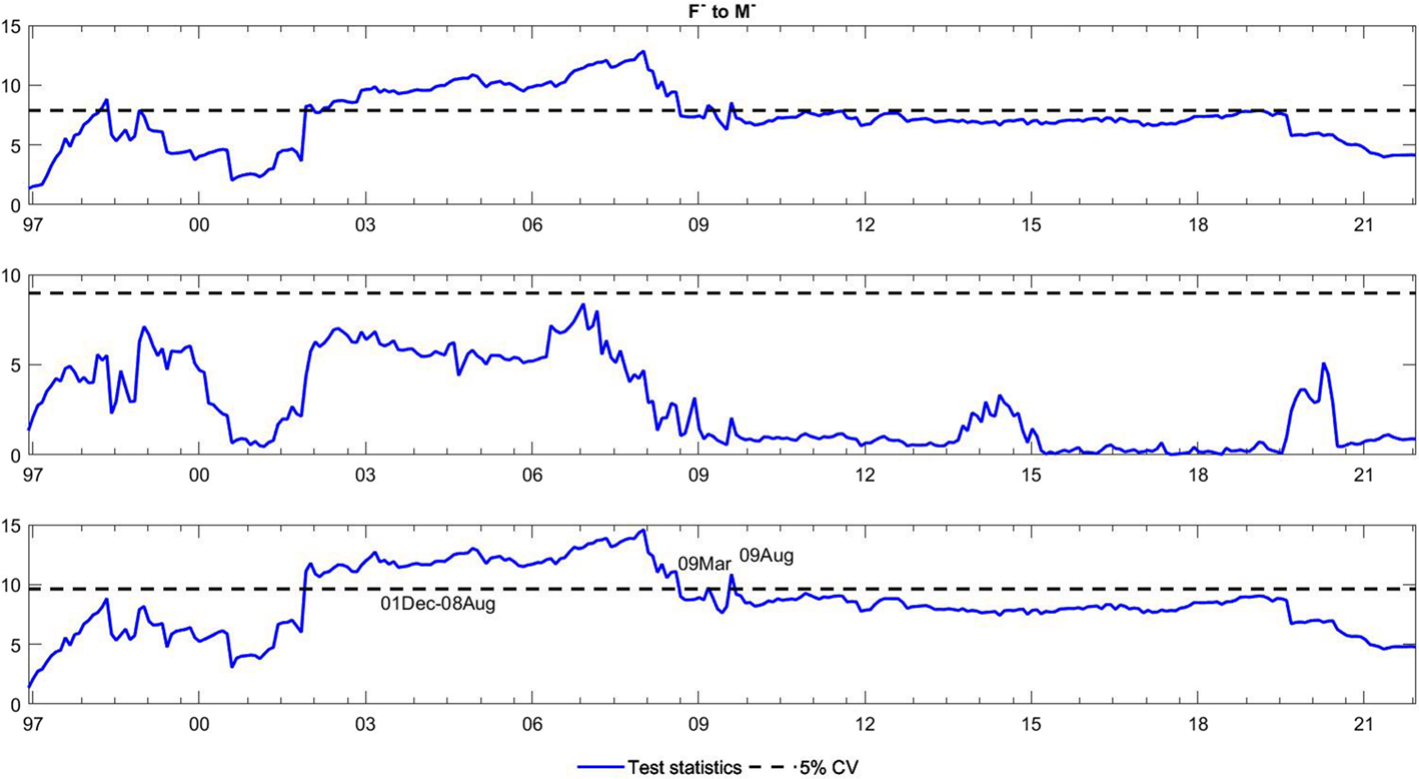
Dynamic causality running from negative FSI shocks to negative MPU shocks. *Notes*: This figure displays the dynamic changes in causality running from ${F}^{-}$ to ${M}^{-}$. If the blue line is higher than the black line, ${F}^{-}$ can Granger cause ${M}^{-}$ (Color figure online)

**Fig. 5:**
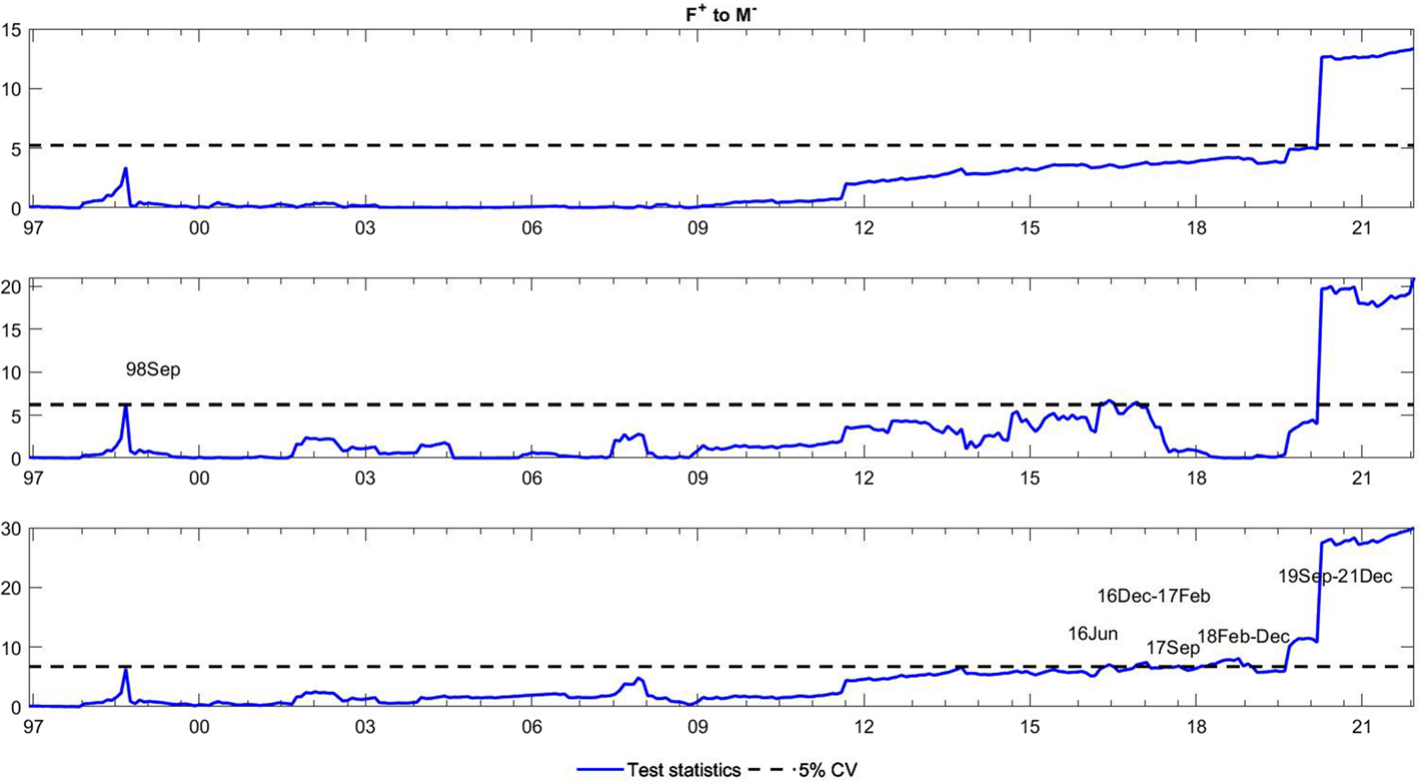
Dynamic causality running from positive FSI shocks to negative MPU shocks. *Notes*: This figure displays the dynamic changes in causality running from ${F}^{+}$ to ${M}^{-}$. If the blue line is higher than the black line, ${F}^{+}$ can Granger cause ${M}^{-}$ (Color figure online)

**Fig. 6:**
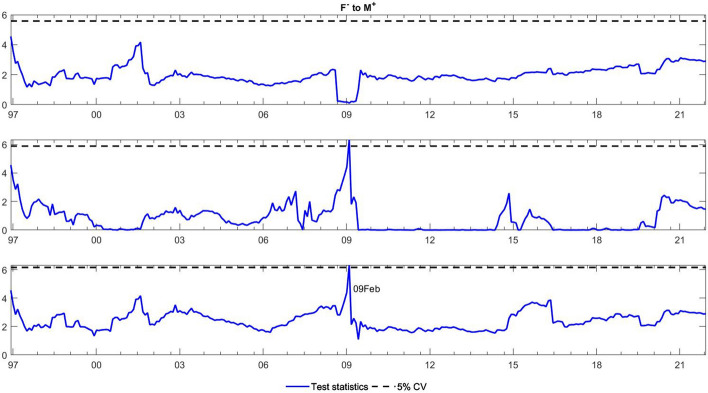
Dynamic causality running from negative FSI shocks to positive MPU shocks. *Notes*: This figure displays the dynamic changes in causality running from ${F}^{-}$ to ${M}^{+}$. If the blue line is higher than the black line, ${F}^{-}$ can Granger cause ${M}^{+}$ (Color figure online)

**Fig. 7:**
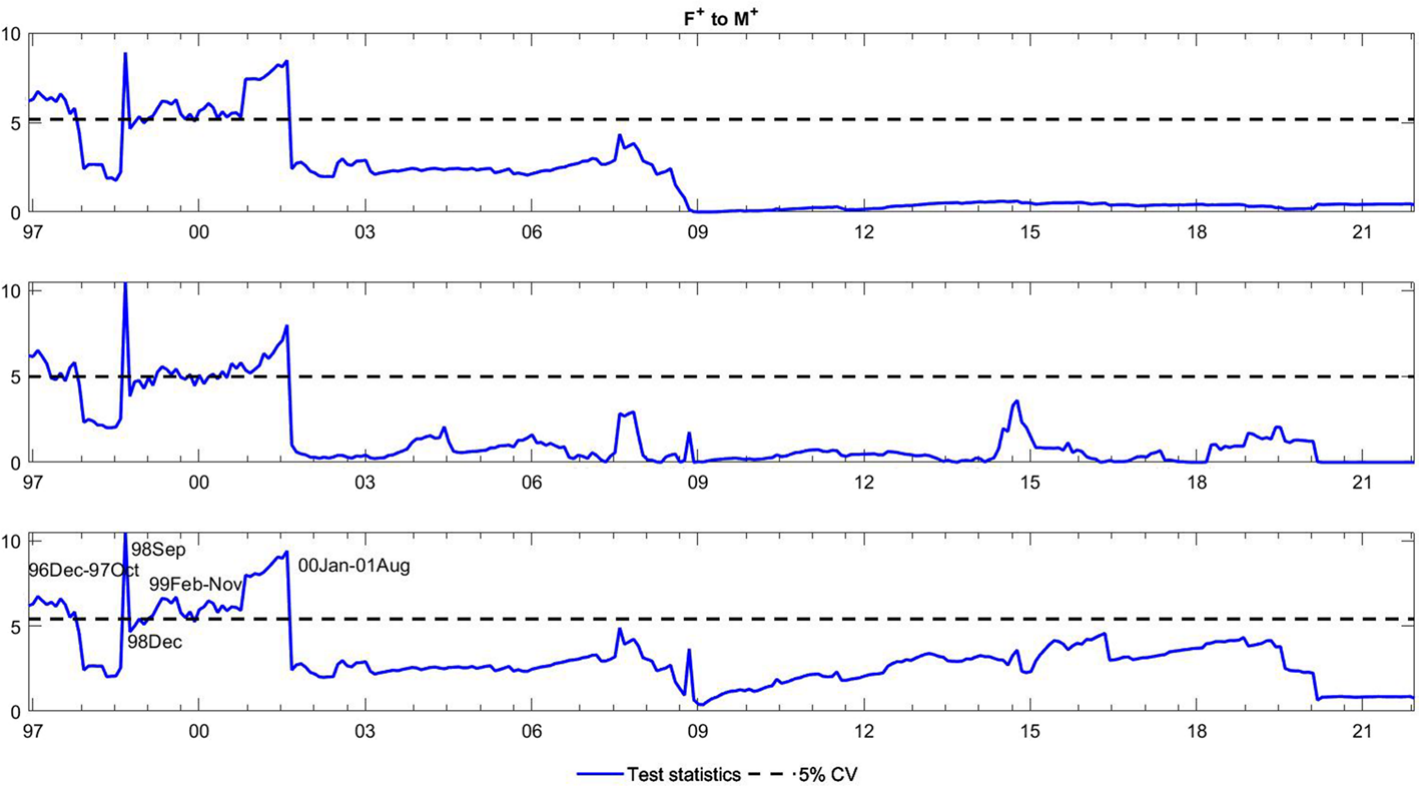
Dynamic causality running from positive FSI shocks to positive MPU shocks. *Notes*: This figure displays the dynamic changes in average causality running from ${F}^{+}$ to ${M}^{+}$. If the blue line is higher than the black line, ${F}^{+}$ can Granger cause ${M}^{+}$ (Color figure online)

**Fig. 8:**
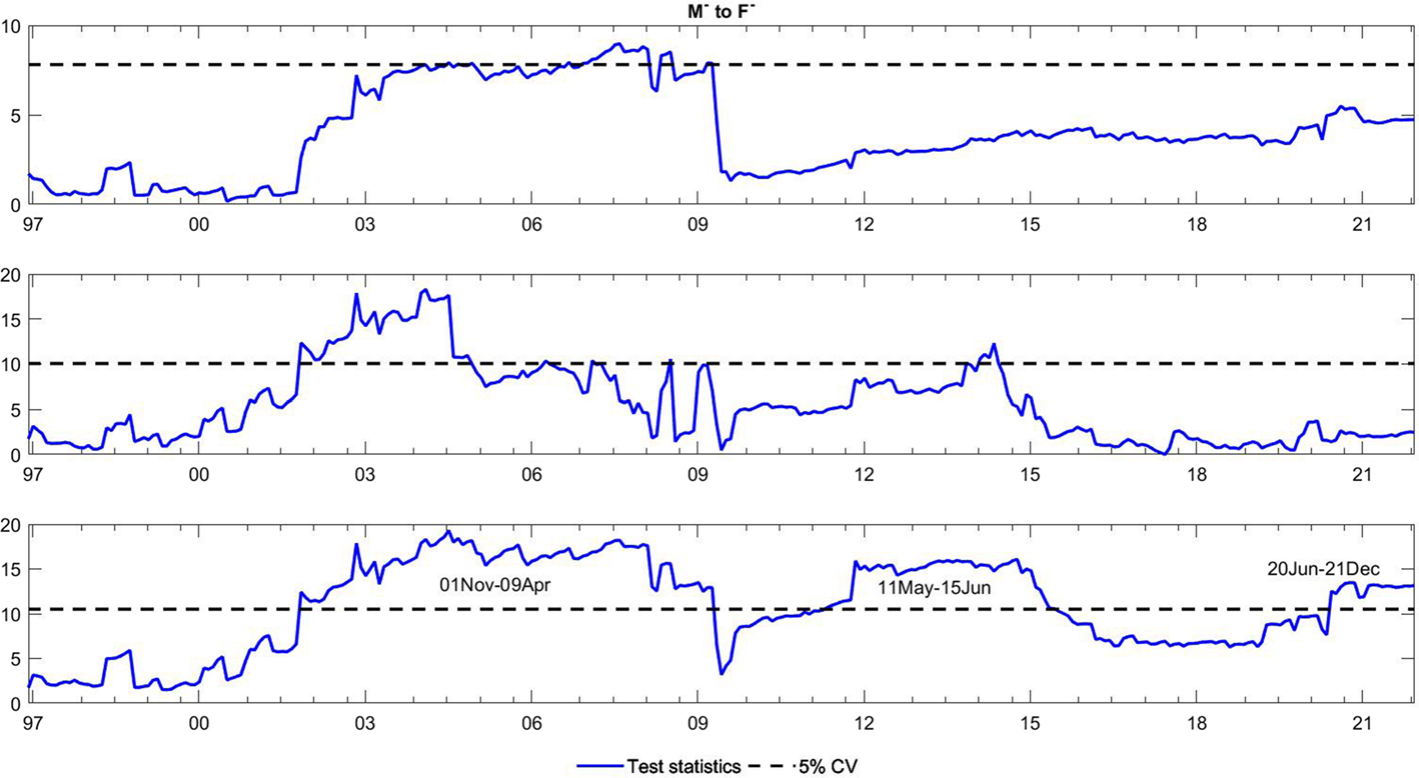
Dynamic causality running from negative MPU shocks to negative FSI shocks. *Notes*: This figure displays the dynamic changes in average causality running from ${M}^{-}$ to ${F}^{-}$. If the blue line is higher than the black line, ${M}^{-}$ can Granger cause ${F}^{-}$ (Color figure online)

**Fig. 9:**
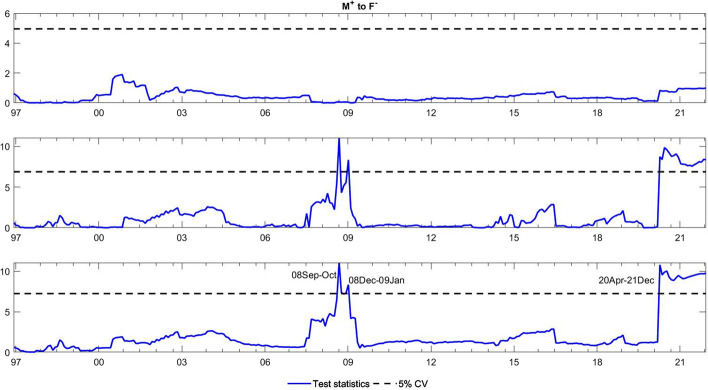
Dynamic causality running from positive MPU shocks to negative FSI shocks. *Notes*: This figure displays the dynamic changes in average causality running from ${M}^{+}$ to ${F}^{-}$. If the blue line is higher than the black line, ${M}^{+}$ can Granger cause ${F}^{-}$ (Color figure online)

**Fig. 10:**
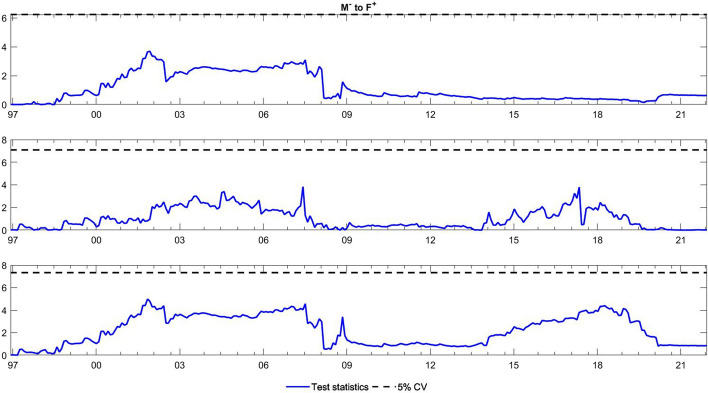
Dynamic causality running from negative MPU shocks to positive FSI shocks. *Notes*: This figure displays the dynamic changes in average causality running from ${M}^{-}$ to ${F}^{+}$. If the blue line is higher than the black line, ${M}^{-}$ can Granger cause ${F}^{+}$ (Color figure online)

**Fig. 11:**
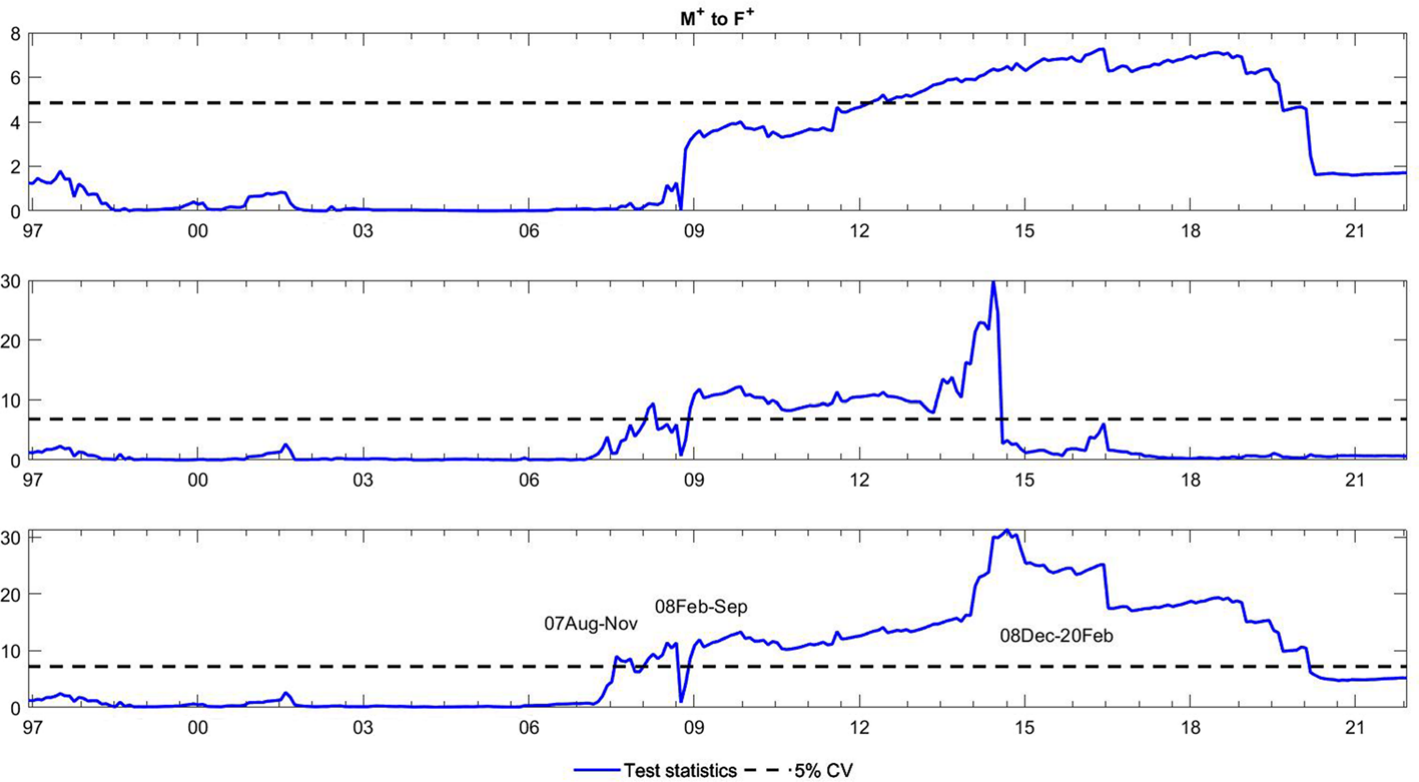
Dynamic causality running from positive MPU shocks to positive FSI shocks. *Notes*: This figure displays the dynamic changes in average causality running from ${M}^{+}$ to ${F}^{+}$. If the blue line is higher than the black line, ${M}^{+}$ can Granger cause ${F}^{+}$ (Color figure online)

To observe the changes in the asymmetric impact of financial stress on monetary policy uncertainty and in the response of monetary policy uncertainty to financial stress, we extend the asymmetric causality test by combining the forward, rolling, and recursive evolving procedures. The empirical results present the dynamic changes in all asymmetric causal relationships between the FSI and MPU. We also show the changes in average causality. In line with Shi et al. (2020) and Hammoudeh et al. (2020), we assume 72 months as the minimum window size and 12 as the maximum lag order. The overall size over a 12-month period is controlled by 5%. The maximum possible order of integration is equal to unity ($d=1$). We mainly focus on the results of the recursive evolving procedure (the third subgraph in each figure). Although the recursive evolving procedure outperforms the other two, we still show the results of the (the first subgraph in each figure) and rolling procedures (the second subgraph in each figure). Figures 2, 3 display the changes in average causality. Figures 4, 5, 6 and 7 show the dynamic changes in causality running from FSI shocks to MPU shocks, while Figs. 8, 9, 10 and 11 show those running from MPU shocks to FSI shocks.

**Q3**: How does the asymmetric causality running from the FSI to MPU change over time? When do structural changes occur?

Figure 2 shows the dynamic changes in average causality running from the FSI to MPU. Interestingly, we see that the results of these three algorithms have similar change trends. We focus on the results obtained from the recursive evolving procedure, which basically identifies significant average causality running from the FSI to MPU from December 1996 to August 2008. Furthermore, we apply the asymmetric time-varying causality test to detect this dynamic change in more detail. For negative MPU shocks, in Figs. 4, 5, we find that negative FSI shocks can affect them during the period from December 2001 to August 2008, while positive FSI shocks can significantly have an impact on them from September 2019 to December 2021. In contrast, in Figs. 6, 7, the null hypothesis of noncausality running from negative FSI shocks to positive MPU shocks cannot be rejected during the whole period, except in February 2009. In addition, positive FSI shocks are effectively predictive factors for positive MPU shocks from December 1996 to August 2001. Thus, the average causality running from the FSI to MPU can be roughly regarded as a combination of the effect of positive FSI shocks on positive MPU shocks and that of negative FSI shocks on negative MPU shocks. In these cases, dramatically increased financial stress may play an important role in determining the positive changes in MPU, while decreased stress can also lead to a falling MPU.

**Q4**: How does the asymmetric causality running from MPU to the FSI change over time? When do structural changes occur?

Figure 3 shows the dynamic changes in average causality running from MPU to the FSI. The results show differences among these three algorithms. We concentrate on the results obtained from the third procedure, which significantly confirmed the average causality running from MPU to the FSI from February 2001 to July 2019. We further make sense of this dynamic change using our novel extended test. Figures 8, 9 visually illustrate how negative and positive MPU shocks affect negative FSI shocks. First, negative MPU shocks can strongly influence negative FSI shocks during three periods, namely, 2001–2009, 2011–2015, and 2020–2021. Moreover, positive MPU shocks can Granger cause negative FSI shocks in two short periods, i.e., from September 2008 to January 2009 and from April 2020 to December 2021. On the other hand, Figs. 10, 11 display the dynamic causality running from negative and positive MPU shocks to positive FSI shocks. Negative MPU shocks cannot affect positive FSI shocks, while positive MPU shocks may provide some useful information for forecasting positive FSI shocks from 2007 to 2020. Therefore, we conclude that the strong causal relationship running from MPU to the FSI is closely related to their negative and positive shocks in different periods. More importantly, effects originating from different shocks may jointly lead to the breakpoint of causality from MPU to the FSI.

## Discussion

Previous research analysing the relationship between financial stress and monetary policy uncertainty has rarely discussed their causal relationship over the past 30 years. In the post-COVID-19 period, various risks are intertwined, which further motivates us to conduct in-depth research on the relationship between financial instability and monetary policy uncertainty. Historically, the reasons for each instance of financial turmoil have been different (Baxa et al. 2013; Adrian & Liang 2016; Bratis et al. 2021). For example, the impact of the COVID-19 crisis on financial instability and monetary policy uncertainty may differ from other major events (Padhan & Prabheesh 2021). From this perspective, this paper fills a gap in the literature. We conduct our analysis by exploring the relationship between their historical fluctuations. If we can uncover the hidden information in historical data, it may provide helpful opinions for policy-makers, institutions and individuals. By employing the asymmetric test, this paper first detects that there is an asymmetric influence between the two financial fundamentals, which means that relevant decision-makers should pay more attention to rising periods of uncertainty. Moreover, we further find that the occurrence of some events may lead to structural breakpoints in the original causal relationship. Therefore, the occurrence of these events may have affected the relationship between them to varying degrees and caused a significant change in this asymmetric effect.

Specifically, increased financial stress reduces uncertainty about monetary policy. When the stability of the financial system is undermined, policy-makers take remedial measures. For example, we take the central bank as an example for analysis.^6^ As one of its important powers, the central bank can adjust the exchange rate. In the case of an imbalance in stability, it will sometimes adjust the exchange rate to some extent to influence monetary policy. Additionally, it needs to be mentioned that central banks do not react by simply changing exchange rates to influence monetary policy, but this behaviour does indeed work in some cases. At this time, monetary policy has become clearer, as it focuses on reducing financial stress. However, Baxa et al. (2013) pointed out that central banks respond only to severely unbalanced changes in asset prices; thus, their responses are asymmetric. In other words, monetary policy changes only when increased financial stress affects asset price movements. In this case, monetary policy uncertainty can be reduced. Conversely, when monetary policy uncertainty rises, financial banks, institutions, and individual investors may prefer to invest in less risky assets to hedge systemic risks. At this point, financial stress may subside.

However, is this situation persistent and unchanging? The answer is not really. From the results of the time-varying test, we find that the asymmetric impact of FSI shocks on MPU shocks and responses of MPU shocks to FSI shocks are dynamic. We observe some crucial breakpoints in Figs. 4, 5, 6, 7, 8, 9, 10 and 11. During our sample period, since 1994, the US macroeconomy has greatly improved and developed due to the rise of new internet-based business models. However, the stability of the financial system began to experience disruptions at this time, leading to high levels of financial stress (Davig & Hakkio 2010). This high stress lasts until the speculative bubble (Dot-Com bubble) burst in 2001. Meanwhile, the formation of a bubble is always accompanied by expansionary monetary policy, and thus, monetary policy uncertainty rose sharply from 1994 to 2001 (Brunnermeier & Schnabel 2015). However, after 2000, in response to the bursting of the dot-com bubble, the Fed lowered the federal funds rate until the subprime crisis of 2007–2008 (Selgin et al. 2015). Hence, between 2001 and 2008, the market tended to be relatively stable with low financial stress. Monetary policy is clear at this time, and thus, uncertainty about it is reduced. This declined uncertainty allows market participants to hold portfolios with the lowest risk, which in turn may contribute to maintaining market stability. During the period after the 2008 global financial crisis, monetary policy was fickle due to the need for economic recovery (Mishkin 2009). These changes led to higher uncertainty, which might affect the behaviour of financial institutions and individuals. They buy and sell more frequently, raising the uncertainty of financial markets (Hoffmann et al. 2013). Notably, the COVID-19 outbreak has been verified to have an apparent impact on financial conditions (Wang et al. 2020a). During this pandemic, the financial system was impacted in many ways, leading to sharply increased financial stress (Corbet et al. 2020). Policy-makers make every effort to stabilize financial stability. In particular, the central bank adjusts monetary policy to achieve this goal, which may potentially decrease the uncertainty about monetary policy. Hence, dynamic changes exist in the causal relationship between monetary policy and monetary policy uncertainty.

## Supplementary analysis

### Nonlinear causality tests

In addition, in actual economic operation, economic variables often have structural mutations due to the impact of economic events such as system changes and financial crises. The resulting system interval effect makes the variables show significant nonlinear characteristics in the process of interaction. However, the traditional Granger causality test examines the linear causal relationship between variables by constructing a linear model and cannot discover the underlying nonlinear causal relationship between variables. Inspired by Sinha et al. (2018), we also used the HJ test (Hiemstra & Jones 1994) and the DP test (Diks & Panchenko 2005) to perform a supplementary analysis of the causal relationship between the FSI and MPU. Diks and Panchenko (2005) pointed out that the HJ test usually does not meet the definition of Granger causality and has the behaviour of excessively rejecting the null hypothesis of non-Granger causality. Therefore, the DP test corrects the over-exclusion problem of the HJ test. However, Yang and Zhao (2014) pointed out that the DP test still over-rejects the null hypothesis to a certain extent. Therefore, the comparison of linear and nonlinear results in this paper can also provide a reference for other researchers. For a better comparison, we also employ monthly sample data from January 1991 to December 2021. Table 5 displays the results of the HJ test and the DP test. These results are half those of Table 4.

**Table 5:** Results of the HJ test and the DP test

${l}_{X}={l}_{Y}$	$F\nrightarrow M$		${F}^{-}\nrightarrow {M}^{-}$		${F}^{-}\nrightarrow {M}^{+}$		${F}^{+}\nrightarrow {M}^{-}$		${F}^{+}\nrightarrow {M}^{+}$	
	HJ	${T}_{2}$	HJ	${T}_{2}$	HJ	${T}_{2}$	HJ	${T}_{2}$	HJ	${T}_{2}$
1	1.0604	1.1440	3.3752***	3.3383***	2.9357***	2.9113***	3.5535***	3.4875***	2.8138***	2.8557***
2	1.2273	1.3383*	2.8282***	2.8197***	2.6186***	2.6067***	3.2706***	3.2007***	2.4713***	2.5237***
3	0.9057	1.0040	2.6407***	2.6579***	2.4154***	2.4094***	3.1180***	3.0770***	2.2448**	2.2997**
4	1.0502	1.2060	2.3127***	2.3476***	2.1043**	2.1029**	2.8479***	2.8294***	1.8607**	1.9200**
5	0.7151	0.8011	1.9647**	2.0068 **	1.9970**	2.0032**	2.5297***	2.5249***	1.7395**	1.7959**

${l}_{X}={l}_{Y}$	$M\nrightarrow F$		${M}^{-}\nrightarrow {F}^{-}$		${M}^{-}\nrightarrow {F}^{+}$		${M}^{+}\nrightarrow {F}^{-}$		${M}^{+}\nrightarrow {F}^{+}$	
	HJ	${T}_{2}$	HJ	${T}_{2}$	HJ	${T}_{2}$	HJ	${T}_{2}$	HJ	${T}_{2}$
1	0.7581	0.7908	1.3864*	1.4458*	1.4104**	1.4695**	1.3722*	1.4072*	1.3171*	1.3435*
2	0.5891	0.5356	1.4204*	1.4767*	1.4135**	1.4723**	1.3463*	1.3799*	1.2608	1.2863
3	0.4548	0.4186	1.4127*	1.4690*	1.4055**	1.4661**	1.2721	1.3044*	1.2125	1.2356
4	0.4832	0.5349	1.4232*	1.4793*	1.4161**	1.4776**	1.2717	1.3042*	1.2115	1.2357
5	0.4392	0.4144	1.4317*	1.4833*	1.4246**	1.4819**	1.2675	1.2979*	1.2100	1.2334

Null hypotheses that are significantly rejected at the 1% and 5% significance level are shown in bold

${l}_{X}={l}_{Y}$ represents the lag order. The results are shown for the HJ test and T_n_ for bandwidth values of 1.5

*, ** and *** denote significance at the 10%, 5% and 1% levels, respectively

In particular, both tests show that we also cannot reject the nonlinear Granger noncausality running from the FSI to MPU, which implies that there is no nonlinear impact of the FSI on MPU. More interestingly, four nonlinear causalities running from FSI shocks to MPU shocks are significantly detected. In particular, both nonlinear tests reveal that the null hypothesis of noncausality running from ${F}^{+}$ to ${M}^{-}$ can be rejected at the significance level of 1%, suggesting that positive FSI shocks play a role in affecting negative MPU shocks during our sample. These findings are in line with the linear test. On the other hand, both tests show the nonexistence of unidirectional causality from MPU to the FSI. In addition, from a nonlinear perspective, the hypotheses of ${M}^{-}\nrightarrow {F}^{-}$ and ${M}^{+}\nrightarrow {F}^{+}$ are not rejected at the 10% level. These results are consistent with Table 4. However, we cannot find evidence of causality running from ${M}^{+}$ to ${F}^{-}$, while we can detect that the null hypothesis of ${M}^{-}\nrightarrow {F}^{+}$ can be rejected at the significance level of 5%. Therefore, MPU may have nonlinear effects on the FSI through its negative shocks.

Hence, this supplementary analysis further demonstrates the importance of accounting for asymmetric shocks. Moreover, comparisons should be made from the linear and nonlinear perspectives to fully explore the potential links between the series when modelling financial time series.

### A kind of proxy for monetary policy uncertainty

Considering the discussion of the central bank’s actions to adjust the exchange rate as an example, this paper takes exchange rate adjustment as a proxy variable for monetary policy uncertainty and uses exchange rate adjustment to explain the relationship between financial instability and monetary policy uncertainty. Motivated by Wang and Lee (2022), we focus on the dollar against four major currencies, namely, the euro, renminbi, Japanese yen, and U.K. pound (hereafter, dollar-euro, dollar-renminbi, dollar-yen, dollar-pound). Based on data availability, the dollar-euro covers the period from January 1999 to December 2021, and the other exchange rates are in line with our main sample.^7^ We provide the results of the asymmetric causality test in Table 6 to better display the impact of the FSI on the exchange rate.

**Table 6:** Asymmetric causality between the FSI and the exchange rate

	Euro	Renminbi	Japanese yen	U.K. pound
*Panel A: FSI ↛ Exchange rate*				
$F\nrightarrow E$	16.8988**	0.0001	1.2374	14.4234**
${F}^{-}\nrightarrow {E}^{-}$	3.5411	1.1394	2.4599	0.1713
${F}^{+}\nrightarrow {E}^{-}$	11.3414**	2.4595	5.7705	15.8934**
${F}^{-}\nrightarrow {E}^{+}$	7.7070**	0.0409	1.9009	0.0035
${F}^{+}\nrightarrow {E}^{+}$	3.9011	0.0059	2.9056	0.2967
*Panel B: Exchange rate ↛ FSI*				
$E\nrightarrow F$	7.9664**	0.0825	2.8831	2.5685
${E}^{-}\nrightarrow {F}^{-}$	11.4224**	1.1707	30.4805**	25.3627**
${E}^{+}\nrightarrow {F}^{-}$	0.0466	0.0180	2.5581	0.2488
${E}^{-}\nrightarrow {F}^{+}$	13.4049**	22.5210**	1.3847	7.5660**
${E}^{+}\nrightarrow {F}^{+}$	0.0231	0.0100	0.6034	0.0417

Null hypotheses that are significantly rejected at the 1% and 5% significance level are shown in bold

*F* stands for the FSI, and *E* is the exchange rate

$+$ and $-$ are positive and negative shocks, respectively

**denotes rejection of the null hypothesis at the 5% significance level

In particular, we find that the bidirectional causality between the FSI and the dollar-euro exchange rate is strongly significant. Financial stress can negatively drive the trend of the dollar-euro exchange rate, while a decreased exchange rate can impact changes in the FSI through its negative shocks. In addition, there is a unidirectional impact of stress on the dollar-pound exchange rate. Positive stress shocks may cause a decrease in the dollar-pound exchange rate, but negative rate shocks may increase financial stress. Interestingly, the null hypothesis of noncausality between the FSI and the other two exchange rates can be rejected. However, we find that the dollar-renminbi can affect an increased FSI through its negative shocks, while the dollar-yen exchange rate can lead to a decreased FSI. Thus, the asymmetric effect on these causalities exists. However, changes in monetary policy are related to the adjustments of the central bank’s different exchange rates, and thus, we cannot analyse the relationship between the FSI and MPU based on a single exchange rate. In summary, we use exchange rate adjustment as a proxy variable for monetary policy uncertainty, which may deepen readers’ understanding.^8^

## Conclusion

Financial system stability is a key factor in promoting economic growth. Instability and uncertainty in the financial system have always been known as financial stress. To the best of our knowledge, many factors contribute to financial system instability. In particular, once this stability is broken, the central bank will revise various current policies to resolve this chaos. Among them, monetary policy is particularly important. However, policy changes in response to a crisis in the short term mean increased policy uncertainty, which may lead to more negative and poor outcomes. This paper pays attention to this uncertainty and aims to fill the gaps in existing research by studying the relationship between monetary policy uncertainty and financial stress. Historically, the causes of each instance of financial turmoil have been different. Whenever such disruptions occur, they have a different impact on financial instability and monetary policy uncertainty from other major events. Therefore, in this paper, we further study the impact of financial stress on monetary policy uncertainty and the response of financial stress to monetary policy uncertainty from the asymmetric and time-varying perspectives.

Based on the analyses above, we first find that the causal relationship between the FSI and MPU is not apparent on average. However, asymmetric causality tests provide evidence of significant causality from positive FSI shocks to negative MPU shocks. This means that increased uncertainty in the financial system reduces monetary policy uncertainty. Conversely, the test results also find that positive MPU shocks can strongly lead to negative FSI shocks, suggesting that increased monetary policy uncertainty can reduce financial stress. Furthermore, we observe dynamic changes in these asymmetric causal relationships by testing asymmetric causal relationships from a time-varying perspective. In particular, we find that different positive and negative shocks may play the main driving role in different periods. Collectively, these structural breakpoints in causality appear to be closely related to several historical economic and non-economic events, notably the dot-com bubble, the 2009 financial crisis, and the current COVID-19 pandemic.

Uncertainty and instability do not equate to a crisis but can spur development. During times of high financial stress, monetary policy uncertainty may be reduced. In particular, some events related to a currency crisis have had a significant impact on the FSI-MPU relationship, such as the expansionary monetary policy caused by the dot-com bubble. Since fixed exchange rates and flexible monetary policies may also lead to currency crises, central banks, institutional managers, and individual investors must pay attention to the subsequent impact of monetary policy changes during periods of increased financial pressure. We also found that financial stress decreases under high monetary uncertainty. After the outbreak of the financial crisis in 2008, the continuous turmoil in the financial market seemed to be related to the increase in MPU, and the change in monetary policy in the early stage of the outbreak of the COVID-19 pandemic also deepened financial pressure. We found that in the early stage of the outbreak, the FSI-MPU relationship was mainly affected by positive MPU shocks, while in the later stage, it was mainly affected by negative MPU shocks. Therefore, policy-makers can formulate some MP-related preventive measures in the early stage of a crisis to address the risks caused by the increase in the FSI, and they should adjust MP appropriately to stabilize the FSI in the late stage of the crisis. Correspondingly, investors can shift their assets to safe-haven investments in the early stage of a crisis and can choose an appropriate time to adjust their investment portfolio in the late stage of the crisis. Therefore, in the current post-pandemic period, both the stability of the financial system and the uncertainty about monetary policy should receive more attention.

## Footnotes

1 The instability of the financial system can also be described by the term “financial stress”, which generally refers to a disruption or shock to the regular operation of the financial market (Hakkio and Keeton 2009).

2 See the section on data for more details on the selection of these indices.

3 Notably, each shock has a potential influence on others.

4 The information criterion: $\mathrm{HJC}=\mathrm{ln}\left(\left|{\widehat{\Omega }}_{j}\right|\right)+j\left(\frac{{n}^{2}\mathrm{ln}T+2{n}^{2}\mathrm{ln}(\mathrm{ln}T)}{2T}\right)$, *j* = 0, …, *p* (Hatemi-j 2003). The Wald test: ${\text{Wald}} = \left( {C\beta } \right)^{^{\prime}} \left[ {C\left( {Z^{\prime}Z} \right)^{ - 1} \otimes S_{U} C^{\prime}} \right]^{ - 1} \left( {C\beta } \right)$.

5 See the above-mentioned website for more details about the difference between the BBD MPU and HRS MPU indices.

6 We take the discussion on the central bank’s exchange rate adjustment behaviour as an example. If exchange rate adjustment is used to explain the relationship between financial instability and monetary policy uncertainty, we choose exchange rate adjustment as the proxy variable of monetary policy uncertainty to conduct a supplementary analysis. See Sect. 6.2 for more details. We would like to thank an anonymous reviewer for this suggestion.

7 Data are retrieved from the FRED; https://fred.stlouisfed.org/.

8 We would like to thank the anonymous reviewers for this valuable comment.
